# How Much do Rural Indian Husbands Care for the Health of their Wives'

**DOI:** 10.4103/0970-0218.39238

**Published:** 2008-01

**Authors:** Amarjeet Singh, Arvinder Kaur Arora

**Affiliations:** Department of Community Medicine, PGIMER, Chandigarh - 160 012, India.

**Keywords:** Pregnancy, safe periods, husband

## Abstract

**Objective:**

To ascertain the response of husbands to their wives' health problems.

**Materials and Methods:**

We selected 100 couples through a systematic random sampling from 4 purposively selected villages; these couples were interviewed by a social worker in rural North India through house-to-house survey. The role of husbands during pregnancy, puerperium and during the wives' illness was explored. Their awareness with regard to the reproductive health problems of their wives was also ascertained. Another outpatient department (OPD)-based interview of female (n = 300) patients was conducted; 50 each at health post, subcenter, primary health centers (PHC), community health center (CHC), 50-bed hospital and apex institution. Previous consultation history was also obtained.

**Results:**

Husbands escorted their wives to hospital in 30–40% cases. This was mainly for a visit to bigger hospitals in cities/towns. Husbands decided regarding the treatment agency in the majority of cases. In 10% cases, they took time off their work during wives' sickness and helped in household work. Consultation within a week was 100%. The husbands' knowledge regarding the safe period was inadequate. Majority (78%) said that women remained ill more often. Most wives were satisfied with the role of their husbands during their pregnancy or illness. A majority (80%) of husbands favored education of women up to the 10th standard and 87% were in favor of working women.

**Conclusion:**

Reasonably favorable attitude of husbands towards their wives' health problems was witnessed. This needs to the carefully nurtured.

## Introduction

Men play important and often dominant roles in making decisions that are crucial to women's reproductive health (RH). Because of the low status of women in the Indian society, they still have to depend on their husbands in order to receive appropriate and adequate health care for their illnesses. Men as a group are frequently blamed for many of women's reproductive health problems, e.g., as a source of sexually transmitted diseases/reproductive tract infections (STDs/RTIs), as a high-risk sexual partner, failing as a chief decision maker in the search for appropriate treatment for their wives and also for failing to act as a responsible partner favoring healthy choices for improving the RH of their wives.(1–3)

Recently, it has been recognized by health planners that men should be focused upon more if the RH of women is to be improved.(4) The ICPD-1994 Cairo Conference had also recognized the importance of men for ensuring women's RH. The conference encouraged a ‘couple’ approach rather than focusing separately on men and women.(1) Against this background, the present study was conducted with following objectives:

- To ascertain the awareness of husbands regarding and their response to wives' health problems.
- To determine the concordance of wives' account of their illness with that of their husbands.

## Materials and Methods

### Health care delivery system in rural India

The rural areas in India are divided into community development blocks (CDBs). There are approximately 500 districts in India, with more than 5000 blocks, to which the government provides health care. Every CDB (average population, 100000) contains 1 community health center (CHC) with 4–5 medical specialists, 3 or 4 smaller primary health centers (PHC) with 2 generalist doctors in a population of 30000 and 20–30 subcenters with 2 health workers per 5000 population. Presently, there are 130983 subcenters in India; 22064 PHCs and 1932 CHCs. The pattern described here varies from state to state.

The survey focused on couples where wives' age was up to 49 years. Each social worker selected 50 houses through systematic random sampling from 4 settings: a nonsubcenter village, a subcenter village, a primary health center (PHC) village and a community health center (CHC) village (Total population of four villages, 12778). These 4 areas were purposively selected from rural field practice area of the department of Community Medicine, Post Graduate Institute of Medical Education and Research, Chandigarh. We aimed to interview at least 100 couples. Keeping in view refusals and noncooperation/nonavailability, the social worker enlisted 120 couples (who indicated their availability for interview) through house-to-house survey. The social worker sought an appointment with the selected respondents for an interview. A pretested/pilot-tested structured interview schedule was used. The social worker first interviewed the women respondents on a fixed day/time. Appointment was again obtained to interview the other spouse (if not available on the same day/time). Both husbands and wives were interviewed separately. In majority of cases, husbands were not available at the time of wives' interview. Hence, they were contacted on subsequent visits. In some cases, the spouse was not available for interview despite earlier commitment. These cases were excluded from the study (20 couples) after 3 unsuccessful visits.

The respondents were asked regarding the role of husbands during ante-, intra- and postnatal care and during the hospitalization of their wives. They were also asked regarding the current illnesses of their wives. Their awareness with regard to the reproductive health status of their wives was also investigated.

In addition to the house-to-house survey, 50 female patients each were interviewed by the social worker in six OPD settings: (i) at health post in a nonsubcenter village where weekly clinic is held by the department, (ii) at a subcenter clinic run by the department, (iii) at a PHC clinic, (iv) at a CHC clinic, (v) at a government 50-bed civil hospital and (vi) at an apex institution i.e., PGIMER, Chandigarh. These patients were enlisted in the waiting area/halls. Random selection was not insisted upon. The available respondents were interviewed consecutively. In civil hospital/PGIMER, various OPDs were included (including the Gynecology/Obstetrics OPD). Another single-page pretested-/pilot-tested structured interview schedule was used for this purpose.

The respondents were asked regarding the nature of their disease, date of onset, consultation lag, money spent, etc. They were also asked (along with the actual observation) regarding the people who had accompanied them to hospital/clinic and the role of their husbands in seeking treatment.

The data was analyzed manually. The responses of husbands and wives were compared. Percentages and chi-square test were used for analysis. Verbatim responses of some of the respondents were also noted on the interview schedule.

### Ethical aspects

All respondents provided their consent. They were explained the purpose of the study. They were informed that the data would be kept confidential.

## Results

### House-to-house survey of 100 couples:

Of the total number of subjects, 19 wives were aged 15–24 years, 46 were 25–34 years and 35 were above 35 years. The literacy rate of husbands was 79% (4 graduates) and that of wives was 59% (2 graduates). Most women (87%) were housewives (6 shopkeepers, 2 in service). Almost half of the husbands (49%) were laborers or farmers (20 shopkeepers, 10 in service). Joint family system was more prevalent (57%). Majority of the families (72%) comprised 5–8 members (13 had 1–4 and 15 had 9–12 members). Half of the couples had 3 or more children. Monthly family income ranged from Rs. 1000 to 15000 (US$ 20–300). Per capita monthly income was Rs. 142–2500 (US$ 3–50). Half of the respondents were lower caste Hindus (52%) and 29% were high caste Hindus. The remaining were Muslims (16%) and Sikhs (3%). Most (92%) belonged to middle or lower middle social class (upper middle class, 3% and lower social class, 5%).

Eleven women were pregnant (4 primigravida) at the time of the survey. One woman became pregnant twice during the study period. In 61 women, the youngest child was more than 2 years old and that in 24 women was less than 2 years old. Of the 36 women (24 + 11 + 1) who became pregnant during the last 24 months, more than 3 antenatal care (ANC) visits were made in 27 (75%) cases. In 14 (39%) cases, husbands escorted the women to the doctor/hospital. Decision regarding the choice of health agency for ANC was taken by husbands (19; 53%), wives (3) or both (6). In 7 cases out of 36 pregnant women, husbands reported regarding the complications during pregnancy (wives reported complications in 10 cases).

Of the 27 women who delivered during the past 24 months (9 were still pregnant when the study ended), 19 were delivered at home by midwives (in 7 cases, doctors and in 1 case, a nurse conducted the delivery in hospital). In all except one (2 as per wives' statement), the mother had a full-term delivery. Some complications were reported in 6 cases (4 according to husbands). Decision regarding the place of delivery was made by the husband in 13 cases (16 according to husbands) and by both husbands and wives in 6 cases.

Household work was resumed by the women within 40 d of delivery in 11 cases as informed by wives (6 according to husbands). In 19 cases, midwives (traditional birth attendants) provided postnatal care. Complications in the postnatal period were reported in 5 cases. In 10 cases, husbands took time off work during pregnancy/puerperium. In all the cases, wives were satisfied with the role played by husbands during pregnancy/puerperium.

Overall, 42 women reported some illness at the time of survey or during the past 15 d as compared to 39 cases where husbands reported their wives to be sick. There was some disparity in the responses of husbands and wives regarding symptoms experienced by the women [Table 1]. Treatment was obtained in all except 4 cases. In 10 cases, consultation was performed on the same day. In the remaining, a doctor was consulted within a week. In most cases, husbands decided regarding the treatment agency. In 15 cases, husbands escorted their wives to the doctor. In 18 cases, the wives visited the doctor alone. In 6 cases, no money and in 15 cases, less than Rs. 100 (∼ $2) was spent on treatment. Thirteen cases spent Rs. 100–500 ($2–11). In 4 cases, Rs. 750 ($17) was spent. Only 2 women reported the use of home remedies. In 7 cases, husbands helped the wives in household work during their illness (in 8 cases, the children did so). In 11 cases, no help was available. In 7 cases, husbands took time off work during wives' illness. All except 3 women said that they were satisfied by their husbands' support during their illness.

**Table 1:** House-to-house survey: Health problems of wives as reported by them and their husbands

Health problems of wives	Respondent	
	
	Husband	Wife
Pain in legs	31	29
Acid peptic disease	21	32
Diarrhea	4	6
Urinary trouble	13	14
Cough (URTI)	22	21
Fever	30	30
Dyspnea	19	29
Diminished visual acuity	23	35
Weakness	44	60
Skin infection/boils	26	27
High BP	64	56
Anxiety/tension	32	39
Insomnia	11	10
Menstrual problem	19	23
Vaginal discharge	28	35
Itching around vagina	5	11
Backache	51	55
Dyspareunia	10	18
Mass coming out of vagina	13	15
Genital ulcer	2	3
Lower abdominal pain	22	22
Headache	37	29
Dental problem	7	7

The figures indicate the number of cases in which health problems were reported.

Half of the women had been hospitalized at least once since their marriage. Thirty-nine women had some major illness at least once since their marriage. Husband was the main decision maker in the selection of the hospital. In 44 of the 50 cases, husband escorted the wife and also stayed with her in the hospital. In 44 cases, the women had undergone some operation. In 5 cases, blood was also transfused. Of them, the husband was the donor in one case. In remaining 4 cases, relatives donated the blood.

Most (83%) husbands informed that in girls, the usual age at menarche was 11–16 years; 11 said it to be 17–20 years. Five were not aware about it (one told it to be 5–10 years). Sixty-six of them told the usual age at menopause to be 40+ years; 25 had no knowledge. A majority informed that the menstrual cycles lasted for 26–30 days. Remaining said it to be 31–35 days (24) or <25 days (2). One had no knowledge about it. The duration of menstrual bleeding was told to be <5 day (50) or 5–10 days (47). Remaining said it to last for 11 days or more. Twenty husbands told that the chances of conception were the highest just after menses, 19 told that midcycle had the maximum risk of pregnancy. One told that the highest risk was during the bleeding days, while the others (8) told that few days before menstruation was the risky period. The remaining had no knowledge about it. Regarding the last menstrual period of their wives (LMP), 78 husbands informed correctly, while 2 had no knowledge about it. Nine gave incorrect information. In 29 cases, menstrual cycles of the wives were irregular. In all except one case, husbands knew regarding the materials used by their wives as menstrual pad. Commercial/market pads were used in 7 women.

Sixty-two couples used some family planning method (tubectomy, 39; copper T, 2; condoms, 17; oral pills, 2; and others, 2). When asked regarding the duration of the use of the family planning methods, in 89 cases, both husbands and wives gave similar responses, while in 11 cases, they gave different responses. Majority (73–74) told that there should be two children in the family (6 opted for 3 children and 20 opted for one child). Most of them (85) told that both husband and wives should decide on family size. Most of them (87–90) said that the gap between children should be 2–4 years. Forty-nine husbands told the correct date of the birth of their children, while 6 gave the incorrect date. Among wives, 57 gave the correct date and 2, incorrect. Remaining did not remember it.

A majority of husbands (78) and wives (83) told that it was women who remained ill more often. Most husbands (75) and wives (67) told that the capacity to tolerate pain and illness was more in women. Fifty three husbands and 37 wives told that the decision regarding treatment should be taken by husbands, while 43 husbands and 61 wives told that the decision should be taken by both. Almost all husbands and wives told that the husbands should escort their wives for treatment.

A majority (78–80) told that wives should be educated up to at least high school. Almost all of them agreed that women should have some money (ready cash) with them. Four husbands said that women should not have any cash. Forty-nine husbands and 55 wives said that women should have Rs. 100–500 ($2–11) with them. Remaining said that they should have more than Rs. 500. Lesser number of husbands (86) as compared to wives (95) were of the view that women should work (13 husbands and 2 wives were against the notion).

Various factors were enumerated by the husbands when asked what was the cause of their wives' illness viz., weakness (21), poor diet (2), tension/worries/stress (20), poverty (3), supernatural (2), tubectomy (6), leucorrhea (7), heat (2), improperly conducted delivery (2), no children (2), death of only son (2) and no male child (2).

Awareness of husbands regarding women's illnesses was less for certain diseases [Table 2].

**Table 2:** House-to-house survey: List of illnesses commonly experienced by women in general as enumerated by the respondents

Women's illnesses	Respondent	
	
	Husband	Wife
Menorrhagia	13	35
Oligomenorrhea	19	41
Dysmenorrhea	1	8
Lower abdominal pain	23	39
Vaginal discharge	26	60
Uterine prolapse	13	42
Low backache	19	35
Swelling of the uterus	4	14
Mass/growth in the uterus	53	43
Abortion	1	7
Uterine carcinoma	22	21
Breast carcinoma	3	4
Genital ulcer	2	2
Blood pressure	11	22
Amenorrhea	6	5
AIDS	15	7
Anemia	20	10
Headache	6	13
Weakness	17	17

### OPD study of 300 women patients

With regard to age, 50 (17%) wives were aged 15–24 years, 114 (38%) were 25–34 years and 136 (55%) were above 35 years. In higher level institutions (civil hospital and PGIMER), consultation was within a month of onset of illness for 36% cases and in 64% cases, consultation was at least one month after the onset. In lower level institutions, these percentages were 56% and 44%, respectively (χ^2^ = 10.7, d.f. = 1; *P* ≤ 0.01). Table 3 shows that the lag between symptom onset and consultation was significantly more for CH/PGIMER as compared to HP, SC, PHC (χ^2^ =2 2.8, d.f. = 2; *P* < 0.001).

**Table 3:** OPD study - Time lag between onset of symptoms & consultation in different health care settings

Consultation lag	Health post/subcentre (n=100)	PHC/CHC (n=100)	Civil hospital/Tertiary hospital (n= 100)
< 1 week	60	47	27
> 1 week	40	53	73

χ^2^ 22.8, d.f. 2, *P* < 0.001

Of the 300 women interviewed, 5 had consulted a doctor within 24 h of their having fallen sick, 59 (20%) did it within 1–7 days. In the remaining, the consultation was at least one week after the onset of illness.

Husbands escorted their wives (81/300; 27%) in more instances where a visit was to the civil hospital or to PGIMER (apex institute). Women's visit to clinic without any escort was more frequent in lower level health facilities. (χ^2^ = 52.0, d.f. = 2; *P* < 0.001). The main reason for husbands not escorting wives to hospital was either the husband was busy (127; 65%) or was out of town (58; 29%). There were more instances (28; 44%) when husbands were not aware of their wives' visit to a doctor for HP-PHC. This was less for CHC-CH-PGIMER visit (4; 24%). Only in 2 cases husbands did not approve their wives consulting a doctor.

Decision regarding consultation of different agencies was taken by wives alone (19; 40%) or by both husbands and wives (107; 36%). However, in PGIMER, it was mostly (35) a joint decision of the couple (only in 3 cases, wives alone decided to consult PGIMER on their own as compared to 31 and 26 cases where wives alone decided for consulting a health post- or subcenter clinic, respectively). In almost half of the cases, some treatment was sought before consulting a particular agency.

Significantly more money was spent when consultation was with higher level medical institutions [Table 4].

**Table 4:** OPD study – Money spent on treatment in different health care settings

Money spent	HP S/C PHC (n=150)	CHC/CH (n=100)	Tertiary / Apex institution (n=50)
<Rs. 100	115	42	12
>Rs. 100	35	58	38

χ^2^ 55.2. d.f. 2. *P* < 0.001

The following were the diagnostic categorization of the patients: High-risk pregnancy (38), abdominal pain (31), vaginal discharge (29), giddiness/headache (25), backache (23), chest pain/URI (19), joint pain (16), scabies/skin infection (18), hypertension (17), weakness (21), gastritis (27), fever (24), menstrual problem (15) and others.

Views of some of the wives and husbands on women's health problems, as recorded during the interviews, are given below.

‘Women discuss their sorrows and miseries with everyone. Men keep their problems to themselves’. (38-year-old male)

‘Men raise a ruckus even if they have a slight headache. Women do all household work even when sick’. (28-year-old female)

‘She is from a rough and tough area-where the *Mahabharata* was fought. It was a dry/barren land. She has similar nature. She can tolerate illness easily’. (25-year-old male)

‘When a woman has some illness, she does not care for her health, instead is worried for other family members’. (21-year-old female)

‘Tolerance depends on the kind of diet one gets. If there is adequate nutritious diet, both (men and women) can tolerate illness’. (30-year-old male)

‘I wanted to have 2 children-one son and one daughter, but my father-in-law wants us to have one more son because there has been only one male child in their family since the last several generations. He wants to break this chain’. (27–year-old female)

‘Women give excuses of various illness only when they get a tolerant husband; they have hundreds of excuses, such as vaginal discharge, swelling of body and operation’. (32–year-old male)

## Discussion

For ensuring optimum RH of their wives, the husbands should have adequate knowledge of the RH problems of women in general.

- Be aware of RH problems of their wives
- Listen to their wives regarding their RH problems
- Take appropriate action for RH problems of their wives, i.e., arrange for appropriate treatment (developing country context).
  ° Escort their wives to hospital
  ° Arrange/provide money for treatment
  ° Permit the wife to seek treatment
- Provide emotional support to wives during their RH problem
- Be understanding to wives' condition during RH problem (empathy)
- Help in household work during RH problem of their wives
- Have healthy attitude toward family planning
- Observe safe sex
- Honor/respect the RH rights of wives

Our study revealed that rural North Indian males were quite concerned with regard to the RH of their wives. They managed well on most of the abovementioned parameters of good husbands. For example, 39% of them accompanied their wives for antenatal check ups. Methodological triangulation through OPD study also endorsed this finding. In most of the hospitalization cases also (44/50), husbands escorted their wives to hospitals and stayed with them. In few cases (10%), they even took time off their duties. In some cases (7), they helped their wives in household work also. Antenatal care (3 visits) coverage was quite favorable (75%). Consultation with a doctor for wives' illness within a week was reported in all cases.

There was, however, ample evidence of expressions of the typical male attitude towards women's health problems.

For example, when asked, ‘Do you help in household work when your wife is sick?’ a husband replied, ‘One can serve food/water to kids. However, serving food to the wife is out of question’ (which means that it is below our dignity).

When asked if he consulted his wife regarding the choice of treatment agency for her health problems, a respondent reported ‘Why to ask wife regarding the treatment. Relief is all she should be concerned about. Women are meant for household work. The families where women's say runs do not prosper. Such families disintegrate soon. Woman is the root cause of all problems’. (27-year-old male)

A majority of husbands had satisfactory knowledge with regard to menstruation. However, misconceptions regarding safe period was widely prevalent. As many as 81% did not have the correct knowledge regarding safe period. Many of them told that the period preceding, during and after the menstrual flow was ‘unsafe’. Similar results were reported in our previous study. Other authors have reported similar findings.(1) Awareness of husbands regarding the menstrual status of their wives was also satisfactory. Most of them told correctly the usual age at menarche in girls. Market pad (sanitary napkin) was used by 7% wives as reported by their husbands. Casual attitude of the husbands regarding family affairs was reflected by the fact that only 49% remembered the date of birth of their children. Some of the husbands were still not in favor of working wives. Male dominance was also reflected by the fact that they were the decision makers in most cases of the choice of treatment agency or place of delivery.

Family and societal customs dictate the extent to which a woman is permitted to seek health care. Mothers-in-law or husbands are the main decision makers as far as health care is concerned. Men and other family members play crucial roles in assuring prompt care. Men are often the ones who decide when a woman's condition is serious enough to seek medical care.(5) Men are often called ‘gate keepers’ because of the many powerful roles they play in society-as husbands, fathers, uncles, religious leaders, doctors, policy makers and local and national leaders. Thus, they can control the access to the health information and services, finances, transportation and other resources. Koenig and Foo had also reported from the rural North India that in the locus of reproductive decision making, wives were likely to occupy a subordinate role relative to their husbands or other family members.(6)

Nevertheless, on the whole, women respondents in our study were satisfied with their husbands' role during pregnancy and puerperium. None of them complained of their husbands. Similarly, all except 3 women were satisfied with the response of their husbands to their illness.

Our study showed that husbands usually do not escort their wives during their illness for consultation with doctors within the village or neighboring health centers. However, for consultation in bigger hospitals, which involved traveling to other towns with presumably more serious health problems, they usually escorted their wives. Our previous studies in a village in Chandigarh and rural Haryana had also yielded similar results.(7,8) Similar views were expressed by Khan et al who opined that men appeared to have a limited role to play in routine illnesses/antenatal care of their wives and that their presence was mainly required during emergencies; RH problems were usually considered women's affairs by their respondents. They opined that perhaps it reflected the social environment, which strictly segregated the roles and responsibilities according to gender and discouraged husband-wife communication particularly on reproductive processes. However, such a scenario helped in sustaining a social structure where physical and moral support to the pregnant women are provided by the entire family rather than the husband alone.(3)

Mulgaonkar *et al.*,(5) reported that husbands often do not permit their wives to go for gynecological check-up. Women often become frustrated and depressed due to the lack of concern demonstrated by husbands for their health and gradually become accustomed to not showing any interest in seeking health care. They summarized their respondents' views that menfolk often felt that women's health needs should be limited to care during pregnancy, childbirth and puerperium. There was no concept of preventive medicine or care for general illnesses or gynecological problems in particular.

As far as husbands' awareness with regard to the status of their wives health is concerned, a comparison of the responses of wives and their husbands regarding enlisting of the RH problems of wives revealed that by and large our male respondents were well conversant with ‘what ails my wife’ status. Khan *et al*, however, had reported considerable inconsistency between the responses of husbands' and wives' on reporting of pregnancy in preceding 24 months. They also reported that at least half of the men were not aware of their wives' health status or the prenatal care services received by them during the previous pregnancy.(3)

In our study, much of the illnesses enumerated by wives matched with those enlisted by their husbands'. Some discordance, however, was noted for few diseases. Some of the symptoms were reported more by wives as compared to their husbands, e.g., acid peptic disease (32 vs. 21), breathlessness (29 vs. 19), weakness (60 vs. 44), diminished acuity of vision (35 vs. 22), dyspareunia (18 vs. 10) and vaginal discharge (35 vs. 28). Symptoms such as tension and irritability/anger were reported more by the husbands for their wives who reported these less frequently.

Similarly, for most of the parameters of RH problems of women, concordance was observed between the responses of wives and husbands. Some discordance was, however, observed for items –such as resumption of work by women after delivery; here, husbands' responses tended to indicate that adequate rest (>42 days) was provided to the wives after delivery (n = 21) as compared to wives who reported that this was not so in all the cases (only 16 women said that they resumed work 42 days after delivery. The remaining began working earlier).

When asked ‘what were the common health problems of women in general’, there was reasonable concordance in the responses of husbands and wives. However, there were some diseases, which were enumerated more by wives, e.g., menstrual problem, abdominal pain, vaginal discharge, uterine prolapse, backache and abortion. Some diseases were, however, enumerated more frequently by husbands as compared to their wives viz., mass in uterus, anemia, AIDS and cancer of the uterus.

Weakness, diet, heat and stress were informed by many husbands as the cause of their wives' illness. Similar findings were observed by the authors in their previous studies.(2,9)

Duration of use of family planning methods as reported by husbands and wives also differed. Others have also reported that men and women differ in the use of the reported contraceptive (men tend to over-report).(10) Reasonably progressive view of husbands regarding the family size was reflected in our study where most of them opted for a two child family with a gap of 2 years in pregnancies.

As far as attitude towards working women is concerned, while most men (86%) agreed that women should work, some (14%) were still against it. Among women, almost all (95%) were in favor of working women. Barring 4 husbands who opined that women should not have any money (ready cash) with them, half of them agreed that they may have cash (<Rs. 500; $10). It was encouraging to note that most husbands and wives agreed that women should be educated up to the 10^th^ standard.

ICPD Cairo (1994) had clearly emphasized the need to involve men in improving the RH status of women.(11) National Population Policy of India, 2000, also emphasized the need to focus on men in IEC campaigns to promote the small family norm.(4) Surveys around the world increasingly are interviewing men and reporting regarding their role in the RH of women.(10) This reflects a widening recognition of the need to focus on men.

Our study also revealed that men, in general, are not as aloof regarding women's (or their wives') illnesses as is generally believed. Although there were expressions of typical male attitude toward the issue related to women's health, some evidence of their favorable attitude toward women empowerment was also witnessed. There is, however, a need of careful nurturing this attitude further by creating an atmosphere conducive to involvement of men in the RH of women. Appropriate, aesthetically devised subtle messages through print and electronic media may also help (e.g., advertisement showing fathers changing the nappies or a husband accompanying the wife to a doctor).
